# Executive Functions and Quality of Classroom Interactions in Kindergarten Among 5–6-Year-Old Children

**DOI:** 10.3389/fpsyg.2020.603776

**Published:** 2020-11-19

**Authors:** Aleksander Veraksa, Daria Bukhalenkova, Olga Almazova

**Affiliations:** Faculty of Psychology, Lomonosov Moscow State University, Moscow, Russia

**Keywords:** preschool age, early childhood education, executive functions, class, quality of classroom interaction

## Abstract

According to international longitudinal studies, the quality of preschool education is of great importance for children’s further development. The modern research’s greatest interest in the field of studying the quality of preschool education is precisely the assessment of the relationship between the teacher and children as well as the teaching quality in kindergarten groups. In this regard, the Classroom Assessment Scoring System (CLASS) seems to be the one of the most relevant for the educational environment quality evaluation. The CLASS methodology (which includes emotional support, classroom organization, and instrumental support) is based on the cultural-historical approach, which shows the interaction between students and adults as the main mechanism for child’s development. The aim of this study is to investigate the relationships between different aspects of the classroom organization quality in kindergarten groups and executive functions components (such as cognitive flexibility, inhibitory control, and working memory) in 5–6-year-old children. The quality of classroom interaction was measured by the CLASS. The study used the Dimensional Change Card Sort (DCCS) method to assess cognitive flexibility and the NEPSY-II subtests “Inhibition” to assess inhibitory control and “Memory for Designs” and “Sentences Repetition” to assess visuo-spatial and verbal working memory, respectively. The study was approved by the Ethics Committee of the Faculty of Psychology at Lomonosov Moscow State University. The study involved 26 kindergarten groups in Moscow. While conducting the research, extreme groups were identified (5 with low quality and 10 with high-quality levels of classroom interaction). Then, three kindergarten groups with low level (65 children) and three groups with high level (68 children) of interaction within classroom were selected and compared. The results revealed that children from groups with low level of classroom interaction have higher results in cognitive flexibility tasks when compared with children from groups with high level of interaction. Also, children from groups with high-quality classroom interaction demonstrated higher results in visuo-spatial working memory tasks and inhibitory control tasks as contrasted with children from low-quality groups. These findings attest to the importance of classroom interaction quality for the executive functions development in the preschool age.

## Introduction

According to international longitudinal studies, the quality of preschool education is of great importance for the further psychological development of children (Vandell et al., 2010; Hall et al., 2013; Hamre et al., 2014; Sylva et al., 2014). Arbitrariness or executive functions are one of the key psychological formations of preschool age and the predictors of the future academic success (Best et al., 2009; Welsh et al., 2010; Willoughby et al., 2012; Diamond, 2013; Yeniad et al., 2013). The significant development of executive functions in preschool age (Vygotsky, 1984; Best et al., 2009; Diamond, 2013) speaks to the importance of examining the factors that are likely to foster this process during this period.

The approaches to understanding arbitrariness are different in Russian and Western psychology. The latter one is traditionally based on the executive functions (EF) model by A. Miyake (Miyake et al., 2000), while the Russian ones refer to the cultural-historical theory of Vygotsky (1984). According to Miyake’s model, EF are a group of cognitive skills that provide targeted problem-solving and adaptive behavior in new situations (Miyake et al., 2000). EF are divided into the following main components: (1) working memory, both visual and verbal; (2) cognitive flexibility, which is related to the ability to switch from one rule to another; and (3) inhibitory control, which presupposes the inhibition of the dominant response in favor of what is required to perform the task (Diamond, 2013). Despite the fact that this model was originally based on the results obtained in adults, the possibility of its use in describing EF development during childhood was confirmed in the works of foreign authors (Lehto et al., 2003; Visu-Petra et al., 2012; Diamond, 2013) as well as Russian researchers (Kiselev, 2016; Veraksa et al., 2018, 2020). Thus, these functions are considered as the basis for a child’s voluntary behavior. The basis of cultural-historical approach is the L.S. Vygotsky conception on the systemic structure of higher mental functions (Vygotsky, 1934). The key point here is the idea that a number of qualitative changes take place in the process of ontogenetic development, which consists of the formation of new systems of functions. Thus, in preschool age, perception is converging with verbal thinking, and a new system is formed as a result of uniting these functions into a single process of reasonable, meaningful perception. By virtue of such a restructuring, a gradual mediation of mental processes occurs with the help of external and then internal means (for example, external and internal speech), which in its turn leads to the formation of voluntary attention, logical memory, speech, thinking, and other higher mental functions (Vygotsky, 1984). Despite the difference in theoretical approaches to understanding preschool development and arbitrariness, the same reality is studied in each of the approaches (Almazova et al., 2016).

Various factors that influence EFs have been described in numerous investigations, such as the child’s individual characteristics: age (Carlson et al., 2013), gender (Wiebe et al., 2008; Sobkin, Veraksa et al., 2016; Veraksa et al., 2020), and non-verbal intelligence (Rueda et al., 2005) as well as different family factors: the family’s socio-economic status (Wiebe et al., 2008; Hook et al., 2013), the parents’ educational level (Ardila et al., 2005), and the quality of parent–child interactions (Hammond et al., 2012). However, the quality of the educational environment has an effect on EF development too. Since learning takes place in the kindergarten while interacting with a teacher and this process affects child’s development (Pianta et al., 2002; Hamre and Pianta, 2007; Kaufman, 2010; Hamre et al., 2013; Hestenes et al., 2015; Duval et al., 2016), traditionally, starting from works of Vygotsky (1934, 1984), it was assumed that the main educational task of the kindergarten in Russia was to help children to acquire different cultural forms of knowledge (e.g., symbols, models, schemes, etc.) as well as cultural tools to operate with them. For a child, an adult is serving as a key figure, a bearer of the ideal forms of culture (social rules and norms of behavior), an example of voluntary actions and cultural means to master his/her own behavior (Vygotsky, 1984). According to Vygotsky, the basic mechanism of development is imitation (Vygotsky, 1984). That is why the figure of an adult is so significant for the implementation of educational programs in Soviet and Russian kindergartens, since all activities there are based on the repetition of a teacher’s actions. However, Vygotsky also stated that the environment is a source of action for a child as it provides opportunity for the activity, approved by an adult (Vygotsky, 1984).

The results of the studies aimed at investigating the influence of the educational environment on the EF stress the importance of a comfortable emotional and psychological climate in a group (Hamre and Pianta, 2007; Hamre et al., 2013; Hatfield et al., 2013). The research devoted to the problem of relations of EF and educational environment shows that stress is one of the main obstructing factors (Schoofs et al., 2008; Hatfield et al., 2013) that is connected with the ability of a teacher to provide emotional support in kindergarten groups. Hatfield et al. (2013) showed that children in classrooms characterized by higher levels of emotional support demonstrate lower levels of cortisol than children in classrooms characterized by lower levels of emotional support. Teachers who take the children’s ideas into account help the latter to feel comfortable in the classroom environment and therefore reduce their level of stress and foster the development of their EFs (Hatfield et al., 2013). A positive teacher–child relationship, that is, a relationship characterized by sensitivity and warmth, leads children to actively engage in learning (Pianta, 1999; Hughes and Kwok, 2007) that affects their EF development (Williford et al., 2013). When teachers meet the needs of the children in their group, children tend to engage more in the activities suggested in the classroom (Hatfield et al., 2013). According to the cultural-historical approach, the social interaction with an adult is the main process that affects a child’s development (Vygotsky, 1984). Furthermore, a child’s engagement in classroom activities helps him/her develop EF while interacting with peers in play and group work (Vygotsky, 2004). According to Vygotsky, “human behavior is a product of the development of a system of social ties and relationships, collective forms of behavior and social cooperation” (38, p. 865).

The results of several studies showed that the quality of classroom interactions, as measured by the Classroom Assessment Scoring System (CLASS) (Pianta et al., 2008), was the predictor of children’s learning ability (Sabol et al., 2013) and EF level (Bodrova et al., 2006; Rimm-Kaufman et al., 2009; Weiland et al., 2013; Hamre et al., 2014). The CLASS method assesses classroom experience in the following three domains: emotional support, classroom organization, and instructional support. Emotional Support refers to a specific teaching behavior that helps children develop warm, supportive relationships, feel comfortable in the classroom, and experience enjoyment about learning and communication. Classroom organization describes a specific teacher behavior that helps children develop skills to regulate their own behavior, maintain interest in learning activities, and get the most learning out of each day in kindergarten. Instructional support refers to a specific teacher behavior that supports children’s cognitive development (Pianta et al., 2008). In the Weiland and colleagues study, it was found that the CLASS parameters (emotional support and classroom organization) are predictors of the EF component development such as inhibitory control, working memory, and switching (Weiland et al., 2013). Hamre et al. (2014) revealed that children from groups with high scores on the instructional support scale showed higher results in passing tests on working memory than children from groups in which scores were low. In groups with high scores on the classroom organization, children showed better results in inhibition task than children from groups with a low score in this domain. The authors suggest that the extensive benevolent nature of positive feedback from an adult is also relevant for EF development (Mashburn et al., 2008). A child’s ability to develop cognitive skills is contingent on the opportunities provided by an adult to express existing skills and scaffold more complex ones (Pianta et al., 2008).

The CLASS methodology is based on developmental theory and the cultural-historical approach ideas, which show the interaction between students and adults as the main mechanism for child’s development and learning abilities (Greenberg et al., 2001; Mashburn and Pianta, 2006; Hamre and Pianta, 2007). Their explanation is based on Vygotsky’s statement of the “zone of their proximal development” that is determined by the content of those tasks that the child cannot yet solve independently but is able to solve in joint activities with an adult (Vygotsky, 1984). From this idea follows the leading role of an adult in the mental development of a child. According to Vygotsky, development processes follow the learning processes (Vygotsky, 1934). Correctly organized teaching relies on the child’s zone of proximal development, on those mental processes that begin to take shape in joint activities with adults and then function in his independent activity. In fact, learning is a communication organized in a special way. That is why the CLASS method is based solely on interaction between teachers and students in classrooms. It does not evaluate the presence of materials, neither other physical environment parameters nor the content of educational program in the kindergarten. This is the main difference of this method from other techniques that assess the educational environment quality, for example, ECERS-R (Harms et al., 2005). Our previous study has shown no significant relationship between EF indicators and the total score on ECERS-R scale and the scores on all seven subscales (Belolutskaya et al., 2018). We assume that these results are due to a confusion between the indicators of the physical environment quality and the quality of staff and children interaction in the kindergarten. Therefore, it is important to separate the teacher–child interaction quality indicators from other parameters of the educational environment. In this regard, the CLASS seems to be the most relevant for the educational environment quality evaluation.

This study is the first Russian research where an attempt is made to compare the children’s EF levels and the quality of the classroom interaction measured by CLASS in preschool kindergartens. In line with previous research (Bodrova et al., 2006; Mashburn et al., 2008; Rimm-Kaufman et al., 2009; Sabol et al., 2013; Weiland et al., 2013; Hamre et al., 2014), it was expected that the quality of classroom interaction in a kindergarten group is significantly related to the EF level of children.

## Materials and Methods

### Participants

The study involved 26 senior groups of public kindergartens in Moscow. The sample of this study consisted of 551 children aged 5 to 6 years [*M* (5.6 years)]. Of these, 271 (49.2%) were boys and 280 (50.8%) were girls. All children were Russian speaking without developmental delays. The children of this age group were chosen because at this age, the differences in the development of EF are the most obvious, whereas by the end of the preparatory group (near 7 years), most children achieve approximately the same level of arbitrariness.

The study included municipal kindergartens in the districts characterized by the same level of infrastructure and designed to accommodate primarily medium-income families. This provided a relatively homogeneous socioeconomic sample. All kindergartens used the same educational program “From birth to school” (Veraksa et al., 2014).

All parents were informed about the aims of the study and gave written consent for children’s involvement in the research.

### Measures

#### Quality of Classroom Interaction

*The quality of classroom interaction* in kindergarten was measured via CLASS (pre-K level) (Pianta et al., 2008). In the United States, the CLASS has been used and validated in thousands of classrooms, from pre-kindergarten to high school (Hamre et al., 2014). This method required at least four 30-min cycles of observation in group (total duration = 120 min/class). The CLASS method assessed classroom experience in three domains: emotional support, classroom organization, and instructional support. These three major domains included 10 dimensions that were evaluated using a 7-point Likert scale. From the points received in each dimension, three domain scores were calculated.

#### Executive Functions

Then, the following four methods were used for the assessment of all the main EF components:

*Inhibition.* The subtest of the NEPSY-II (Korkman et al., 2007) “Inhibition” was used for the inhibition assessment. The stimuli included two pages depicting black and white squares and circles. One page, used for practice, displayed eight figures arranged in a line; the other page, used for test trials, displayed 40 figures arranged in five lines. The task consisted of two parts: (1) naming (the shape of each object depicted on the page) and (2) inhibition (naming each object with the opposite name – i.e., saying “circle” when seeing a square). Every child was instructed to complete the page as quickly as possible. The number of errors (both corrected by a child or not corrected) and time spent for the execution of both tasks were recorded.

*Cognitive flexibility.* The Dimensional Change Card Sort (Zelazo, 2006) was used to assess the cognitive flexibility. Children were required to sort a series of bivalent test cards (with pictures of red rabbits and blue boats), firstly according to one dimension (color) and then according to another (shape). At the third try, a child had to sort cards according to the more complicated rule with the additional factor (cards with/without borders). The accuracy score was calculated (max = 24).

*Verbal working memory*. The “Sentences Repetition” subtest of NEPSY-II was used to assess the verbal working memory. The stimuli included 17 sentences of increasing length and complexity. One sentence at a time was read aloud to every child and then the child was asked to repeat it. If a child recalled the sentence correctly, the response was scored with 2 points; if there was one or two mistakes, the response was scored with 1 point; in case there were more than two mistakes, the response was scored with 0 point. If a child received 0 point on three consecutive trials, the procedure was stopped; otherwise, a child received all 17 sentences of the task. Then, the accuracy score was calculated (max = 34).

*Visual working memory*. The “Memory for Designs” subtest of NEPSY-II was used to assess the level of visual working memory and the visual-spatial orientation. This task included four trials. On each trial, every child was shown a grid with four to eight designs. The grid was displayed for 10 s and then taken away. Next, the child was provided with a blank grid and a set of cards, some of which depicted the same designs that were presented before. The child’s task was to select the appropriate designs and place them on a grid in the same location as previously shown. In this test, the following total scores were recorded: (1) “Content,” which reflects the correctness of memorizing the image details, (2) “Spatial,” which reflects the correctness of remembering the configuration, and (3) “Bonus,” which stands for the correct memorization and consideration of both parameters simultaneously. Finally, all three indicators were summarized in the total score (max = 120).

The results of a study on approbation of this battery of methods showed its reliability and relationship with the results of traditional Russian qualitative methods (Almazova et al., 2016). These four methods, in contrast to traditional Russian assessment tools (Venger and Kholmovskaya(eds.), 1978), allow us to see not only qualitative but also quantitative differences in the development of arbitrariness. They are more reliable, less subjective, and more appropriate for the statistical analysis. In the previous research, the most revealing methods on arbitrariness assessment were identified as follows: the “Inhibition” test (NEPSY-II) in the case of A. Miyake model and methods of “Schematization” in the abilities theory of Venger (1986).

As an additional control measures of children development, we used age, sex, and non-verbal intelligence level. Non-verbal fluid intelligence was assessed with the Raven’s Colored Progressive Matrices test (Raven et al., 1998). The task included three sets of matrices, 12 items per set. Each item presented a pattern of geometric designs with a missing piece. The task was to pick the missing piece from six available options. Children were tested individually with no time limit, but the procedure was stopped when the child responded incorrectly on four items in a row. Accuracy scores were calculated (max = 36).

### Procedure

The quality of classroom interaction assessment was conducted during the school year by one certified specialist in CLASS method.

The EF assessment was performed in the second half of the school year, during two individual meetings with each child (every meeting lasting 20–25 min), in a quiet room of the child kindergarten. Children were explicitly asked about their desire to participate in the research and were free to quit or refuse to participate in the research at any time as well.

Feedback on the results of the CLASS assessment was provided to the kindergarten administration together with the generalized results of the children assessment at the end of the school year.

## Results

The descriptive statistics (means and standard deviations) for CLASS and EF measures are presented in Tables 1, 2. The CLASS domain and dimensions scores are mostly fluctuating around the medium quality level (4–5 scores) (see Table 2). In Moscow kindergartens, teachers are good at creating emotional support (*M* = 5.45) and in organizing group work (*M* = 5.12), while instrumental support in most groups is at a low level (*M* = 3.18). The highest results were obtained for the following dimensions: teacher sensitivity (*M* = 6.10) and negative climate (*M* = 1.98 – it is reverse scale) in the emotional support domain and behavioral management (*M* = 5.58) and productivity (*M* = 5.27) in the classroom organization domain. The lowest results were obtained in the concept development dimension (*M* = 2.60) in the instructional support domain.

**TABLE 1:** Descriptive statistics for EF and non-verbal intelligence measures (means and standard deviations).

	**Mean**	**St. dev.**
Non-verbal intelligence	13.25	7.03
Verbal working memory	18.42	4.73
Visual working memory	74.75	21.43
Naming time	47.06	12.45
Naming uncorrected errors	0.76	1.84
Naming corrected errors	1.01	1.18
Inhibition time	63.45	19.05
Inhibition uncorrected errors	3.44	6.69
Inhibition corrected errors	2.15	1.93
Cognitive flexibility	18.88	2.95

**TABLE 2:** Results of CLASS domains and dimensions assessment (means and standard deviations).

	**All groups**		**Low CLASS level**		**High CLASS level**	
	**Mean**	**St. dev.**	**Mean**	**St. dev.**	**Mean**	**St. dev.**
Positive climate	5.08	1.26	3.33	0.67	5.37	1.46
Negative climate	1.98	1.07	3.01	1.91	1.34	0.48
Teacher sensitivity	6.10	0.91	4.45	0.41	6.49	0.71
Regards for students perspectives	4.59	1.06	3.07	0.45	5.34	0.09
Behavioral management	5.58	0.73	5.35	0.67	6.35	0.63
Productivity	5.27	1.00	4.03	0.66	6.28	0.04
Instructional learning formats	4.50	1.12	3.39	0.57	5.63	0.10
Concept development	2.60	1.23	2.22	0.72	2.89	0.30
Quality of feedback	3.39	1.07	2.90	0.68	3.81	0.41
Language modeling	3.54	0.87	3.10	0.60	4.02	0.33
Emotional support	5.45	0.86	4.23	0.64	6.03	0.50
Classroom organization	5.12	0.85	4.01	0.78	5.71	0.46
Instructional support	3.18	1.00	2.60	0.49	4.16	0.64

At the first step of statistical analysis, the cluster analysis of the results in the CLASS assessment was carried out (using the K-means method). It allowed us to identify three groups with low, medium, and high levels of teacher–child interaction (Table 3). All groups have significant differences in all CLASS domains (the Kruskal–Wallis criterion, *p* ≤ 0.005).

**TABLE 3:** Final centers of clusters based on the three CLASS domains.

**CLASS domains**	**Low level (*n* = 5)**	**Medium level (*n* = 11)**	**High level (*n* = 10)**
Emotional support	4.2	5.5	6
Classroom organization	4	5.1	5.7
Instructional support	2.6	2.6	4.2

At the second step, the extreme groups were identified: 5 with low and 10 with high levels of classroom interaction quality. Three kindergarten groups with low quality level (65 children) and three groups with high quality level (68 children) were selected. We selected these groups in pairs so that they were from the one kindergarten (that is, they have the same level of physical environment), but they differed in the quality of interaction. Groups with high level of teacher–child interaction had significantly higher level of the emotional support, classroom organization, and instructional support than groups with low level of classroom organization.

The pair-wise comparison of selected groups with low and high quality level of classroom interaction showed significant differences (the Mann–Whitney test, *p* ≤ 0.001) in all 10 dimensions of the CLASS method (see Table 2).

The pair-wise analysis of the groups with low and high quality levels of classroom interaction (see Table 4) did not reveal statistically significant differences in age, non-verbal intelligence, and verbal working memory. In addition, cross-tab analysis showed that there were no significant differences in the number of girls and boys in the selected groups (in the group with low CLASS level, there were 50% of boys, and in the group with high CLASS level, 57% were boys).

**TABLE 4:** The differences in age, non-verbal intelligence, and executive functions components in preschoolers with low and high quality of classroom interaction.

	**Low CLASS level**		**High CLASS level**		**Mann–Whitney test**	
	**Mean**	**St. dev.**	**Mean**	**St. dev.**	***U***	***p*-value**
Age	67.71	3.90	67.15	4.40	2,065.500	0.426
Non-verbal intelligence	14.11	10.09	14.44	6.95	1,997.000	0.337
Verbal working memory	19.48	5.44	18.90	5.11	1,935.500	0.216
Visual working memory	68.33	20.38	80.65	20.40	1,122.500	0.001
Cognitive flexibility	18.92	2.71	17.79	2.97	1,653.000	0.011
Naming uncorrected errors	0.70	1.23	0.47	1.08	1,688.000	0.025
Naming corrected errors	1.13	1.36	1.08	1.17	2,066.000	0.949
Naming time	45.51	13.10	43.17	9.37	1,835.500	0.251
Inhibition uncorrected errors	3.21	6.43	1.55	3.38	1,531.500	0.005
Inhibition corrected errors	2.08	2.24	2.59	1.97	1,669.000	0.050
Inhibition time	60.86	19.20	58.85	14.46	2,040.500	0.856

However, children from the groups with high level of classroom interaction have significantly higher results in visual working memory test and make less mistakes in “inhibition subtest” than children from the groups with low level of classroom interaction. At the same time, children from the groups with low CLASS level better perform the cognitive flexibility task than children from the groups with high CLASS level.

Thus, the analysis showed the presence of significant differences in the level of executive functions development in groups of children with low and high quality of the classroom interaction.

## Discussion

This study aimed to examine the relationship between the quality of classroom organization in kindergarten groups and the executive functions level of preschool children. As a result of comparing the two extreme groups, significantly varying in the level of classroom interaction in the group, crucial differences were revealed in the development of visual working memory, cognitive flexibility, and inhibition.

The results of this research indicate that children from groups with high quality of classroom interaction have a higher level of development of visual working memory. It is important to note that the memory-for-designs technique used in the study makes it possible to assess not only the level of development of visual working memory and spatial orientation but also the children’s learning ability. Therefore, it is possible to conclude that in groups with a high quality environment, children remember visual information more easily as well as learn more easily. This is also confirmed by the results of previous studies (Weiland et al., 2013).

Unlike previous studies (Weiland et al., 2013; Duval et al., 2016), no relationship was found between verbal working memory and the quality of the classroom interaction. In the classroom, as shown by Ornstein et al. (2010), the teacher’s language during instruction has an effect on the children’s memory development. The absence of significant differences in this study may be due to the peculiarities of the methodology that has been used, the success of which is associated with the language development of children (Klem et al., 2015), while in other studies, less-associated-with-speech tests were used (for example, The Forward and Backward Digit Span method).

As a result of the conducted analysis, it was found that in the inhibition task, children from groups with a high quality of environment make fewer uncorrected errors and slightly more corrected ones than children from high-quality groups. This being said, the time spent for completing this task in the two groups did not differ significantly. Consequently, in groups with a high quality of classroom interaction, children have acquired better skills in cognitive control and have more developed skills for self-examination when performing tasks. This finding is consistent with other studies showing that EF skills develop through social interactions (e.g., Pianta, 1999; Williford et al., 2013). It is in joint activities with the teacher and peers that the preschooler develops his/her ability to inhibit control.

On the other hand, the research results revealed that the level of cognitive flexibility was higher in the groups with low level of classroom interaction. This finding can be explained by the peculiarities of the arrangement of work in groups: according to the CLASS method, a high level of arrangement of work in a group presupposes that children clearly know and follow all the rules and daily routine of the group – they act like a “well-oiled machine” (Pianta et al., 2008). In such a group, the child rarely has to adapt to new requirements and conditions, which possibly slows down the development of cognitive flexibility. Whereas in groups where classroom interaction is less well organized, children have to constantly switch between different rules and sudden events, which contributes to the training of cognitive flexibility. A more detailed analysis of the relationship between individual domains and dimensions of the CLASS methodology with the results of cognitive flexibility technique would make it possible to clarify the obtained result and find out which of the interaction parameters has the greatest impact on the development of this ability.

### Strengths, Limitations, and Suggestions for Future Research

Importantly, in the selected extreme groups, children did not differ significantly based on a number of factors influencing the level of EF development such as the following: the level of non-verbal intelligence development (Rueda et al., 2005), age (Carlson et al., 2013), and gender (Wiebe et al., 2008; Sobkin, Veraksa et al., 2016; Veraksa et al., 2020). This suggests that the results obtained are not related to the impact of these variables. It is also important to note that the analyzed groups with low- and high-quality interaction were selected from the same kindergartens. This emphasizes the importance of the teacher–child interaction quality in the EF development and not the quality of the kindergarten’s physical environment. Thus, the study showed that it is the teacher-to-children communication as well as the teacher’s ability to facilitate communication between children that is most closely related to the development of the EF in preschoolers. The result obtained confirms the provisions of the cultural-historical approach about the significant role of an adult in the development of a child (Vygotsky, 1934, 1984). Throughout the teacher–child interaction occurs the internalization of cultural norms and means of voluntary behavior (Vygotsky, 1984, 2005). Therefore, it is the use of the CLASS method (Pianta et al., 2008), which is focused on interaction, in contrast to the ECERS-R (Harms et al., 2005), which connects us to cultural-historical theory.

However, this study still has a number of significant limitations. The analyzed sample consisted of only 132 children from six kindergarten groups that does not allow the researchers to generalize results. Moreover, in this investigation, family factors were not taken into account. For example, the parents’ educational level and the quality of parent–child interactions both have an effect on executive functions development (Rueda et al., 2005; Hook et al., 2013; Almazova et al., 2016).

In a year, it is planned to retest EF in this sample and to enhance the study with the information on the parents’ educational level, which will make it possible to analyze the contribution of the quality of interaction in the kindergarten to the development of EF while also controlling family factors. In addition, we plan to increase the sample, which will allow us to further analyze the individual contribution of the CLASS domains to the preschoolers’ EF development.

## Conclusion

As a result of the study for the first time in a Russian sample, a relationship was established between the quality of interaction in kindergarten groups and the development of EF components in children aged 5–6 years. The results obtained show the importance of such teacher’s skills for children’s development as creating an emotionally pleasant atmosphere in the group, organizing the group work of children, and using effective techniques for the development of preschoolers’ thinking and speech.

Thus, the CLASS method has shown itself to be an important tool in measuring the quality of the educational environment of a kindergarten.

## Data Availability Statement

The raw data supporting the conclusions of this article will be made available by the authors, without undue reservation.

## Ethics Statement

The studies involving human participants were reviewed and approved by the Ethics Committee of the Faculty of Psychology at Lomonosov Moscow State University (the approval No: 2018/41). Written informed consent to participate in this study was provided by the participants’ legal guardian/next of kin.

## Author Contributions

AV contributed to the presented idea (i.e., research questions) and the theoretical framework. DB was involved in data collection. OA verified the analytical methods and conducted the analyses. DB wrote the manuscript with critical feedback and input from AV and OA. All authors discussed the results and approved the submitted manuscript.

## Conflict of Interest

The authors declare that the research was conducted in the absence of any commercial or financial relationships that could be construed as a potential conflict of interest.
